# Impact of Missense Mutations on AFB1 Metabolism in Bovine Cytochrome P4503A Isoforms: A Computational Mutagenesis and Molecular Docking Analysis

**DOI:** 10.3390/ijms252312529

**Published:** 2024-11-22

**Authors:** Ludovica Montanucci, Silvia Iori, Maija Lahtela-Kakkonen, Marianna Pauletto, Mery Giantin, Mauro Dacasto

**Affiliations:** 1 Department of Neurology, McGovern Medical School, UTHealth—University of Texas Health Science Centre at Houston, Houston, TX 77030, USA; ludovica.montanucci@uth.tmc.edu; 2 Department of Comparative Biomedicine and Food Science, University of Padua, 35020 Padua, Italy; silvia.iori@unipd.it (S.I.); marianna.pauletto@unipd.it (M.P.); 3 School of Pharmacy, University of Eastern Finland, 70210 Kuopio, Finland; maija.lahtela-kakkonen@uef.fi

**Keywords:** cytochrome P450, CYP3A, aflatoxin B1, nifedipine, cattle genetic variants, single nucleotide variants, Piedmontese cattle breed, molecular docking, computational mutagenesis

## Abstract

Cytochrome P450 3A (CYP3A) enzymes catalyze the metabolism of a wide range of endogenous and exogenous compounds. Genetic variations in the 3 CYP3A isoforms (CYP3A28, CYP3A74, and CYP3A76) may influence their expression and activity, leading to inter-individual differences in xenobiotic metabolism. In domestic cattle, understanding how genetic variations modulate CYP3A activity is crucial for both its therapeutic implications (clinical efficacy and adverse drug effects) and food safety (residues in foodstuff). Here, we updated the variant calling of CYP3As in 300 previously sequenced Piedmontese beef cattle, using the most recent reference genome, which contains an updated, longer sequence for *CYP3A28*. All but one previously identified missense variants were confirmed and a new variant, R105W in CYP3A28, was discovered. Through computational mutagenesis and molecular docking, we computationally predicted the impact of all identified CYP3A variant enzymes on protein stability and their affinity for aflatoxin B1 (AFB1), a potent carcinogen and food contaminant. For CYP3A28, we also computationally predicted its affinity for the probe substrate nifedipine (NIF). We found that CYP3A28 with R105W variant cannot accommodate NIF nor AFB1 in the binding pocket, thus affecting their metabolism. Our work provides computational foundation and prioritized ranking of CYP3A variants for future experimental validations.

## 1. Introduction

Cytochrome P450 (CYP) enzymes are a superfamily of heme-containing monooxygenases that catalyze the oxidative metabolism of a wide range of endogenous compounds, including steroids, bile acids, and xenobiotics [1]. Among these, the human CYP3A stands out as one of the most important subfamilies due to its predominant role in the phase I (oxidative) metabolism of over 50% of all therapeutic drugs [2,3]. The human CYP3A subfamily comprises four isoforms: CYP3A4, CYP3A5, CYP3A7, and CYP3A43, which are located on chromosome 7 [4].

The activity of CYP3A enzymes can be influenced by several factors, including exposure to enzyme inducers (e.g., anticonvulsant medications and rifampicin) and inhibitors (e.g., azole antifungal agents and macrolide antibiotics), which can significantly modulate the CYP3As metabolic function [5]. However, mounting evidence suggests that genetic variations within *CYP3A* genes also significantly contribute to the inter-individual variability in enzyme expression and activity, which may lead to differences in drug metabolism and adverse drug reactions. For instance, the CYP3A5*3 allele results in a complete loss of function [6], while the CYP3A4*22 allele results in reduced mRNA expression and lower CYP3A4 enzyme activity [7,8,9]. These genetic differences may play a critical role in determining individual responses to drugs, including susceptibility to drug toxicity and altered pharmacokinetics.

Although pharmacogenetics, the study of how genetic variations influence individual responses to drugs, has made significant advancements in human medicine, pharmacogenetic research in the veterinary field—particularly in the context of veterinary food-producing species—remains in its early stages, despite the critical role these animals play as sources of food products for human consumption. Livestock are regularly exposed to veterinary drugs, pesticides, feed additives, and natural contaminants. The presence of these substances and their metabolites in animal-derived food products, such as meat, milk, and eggs, raises significant concerns for human health. A major threat is posed by the carcinogenic potential of certain compounds, including mycotoxins (e.g., aflatoxin B1—AFB1) and veterinary drugs such as nitrofurans, which can form stable, long-lasting residues in animal products [10]. For instance, AFB1 is metabolized in livestock to AFM1, a compound commonly found in milk and dairy products, and it has been associated with hepatocellular carcinoma in humans after prolonged exposure [11,12]. Another concern is the potential impact of pesticides, hormones, and certain veterinary drug residues classified as human endocrine disruptors, which can interfere with hormonal balance and regulation, posing risks of reproductive health issues, developmental delays, and metabolic disorders [10]. Therefore, understanding the genetic basis of CYP-mediated metabolism in these species is essential for ensuring food safety and minimizing harmful residues in animal-derived food products, as well as improving knowledge of the clinical efficacy and potential adverse effects of drugs.

Cattle (*Bos taurus*) are one of the most important farm animal species and a significant source of nutrition worldwide. Three bovine *CYP3A* genes have been annotated on chromosome 25 (genome ARS-UCD1.2): *CYP3A74* (also known as *CYP3A4-like*, previously recognized as *CYP3A28*; Ensembl Genome Browser ID ENSBTAG00000052665); *CYP3A76* (also known as *CYP3A5-like*, before known as *CYP3A38*; ENSBTAG00000053645); *CYP3A28* (also known as *CYP3A4* nifedipine oxidase, earlier identified as *CYP3A48*; ENSBTAG00000049666) [13,14,15,16].

Previous studies investigated the role of the bovine CYP3A subfamily in the biotransformation of therapeutic drugs and xenobiotics, specifically its involvement in the metabolism of the antiparasitic macrocyclic lactone moxidectin, the ionophore antibiotic monensin, as well as tiamulin and macrolide antibiotics [17,18,19]. Furthermore, recent reports indicate that some acaricides and organophosphate compounds, commonly used for controlling ectoparasites in livestock and in protecting crops, can inhibit CYP3A-dependent enzymatic activity in bovine liver microsomes [20]. This inhibition may decrease the CYP3A-mediated metabolism of concurrently administered drugs, potentially leading to harmful drug–drug interactions.

Moreover, in our recent study, we compellingly demonstrated that the bovine CYP3A74 isoform plays a crucial role in the oxidative metabolism of aflatoxin B1 (AFB1), a globally significant mycotoxin [21]. In mammalian and avian species, hepatic CYP3A catalyzes the 8,9-epoxidation of AFB1, which can result in the formation of the AFB1 8,9-*exo*-epoxide (AFBO) derivative, which can bind to DNA, triggering mutations and carcinogenesis [22,23]. CYP3A also facilitates hydroxylation reactions, leading to the production of the genotoxic AFM1 (the C9-hydroxylated metabolite of AFB1) and the less harmful aflatoxin Q1 (AFQ1, the C3-hydroxylated metabolite) [24]. Our study specifically highlighted the prominent role of the CYP3A74 isoform in forming the AFB1 8,9-*exo*-epoxide metabolite [21]. When dairy cows consume AFB1-contaminated feed, detectable amounts of AFM1 derivatives can be found in milk, posing a potential health risk to humans [25,26]. According to EFSA guidelines, AFM1 concentrations in milk exceeding 0.05 µg/kg are considered unsafe [22], as they significantly heighten carcinogenic risks for consumers. Additionally, exposure to AFB1 in cattle can impair liver function, weaken immune responses, and reduce feed intake, which collectively can affect growth and milk production and increase susceptibility to diseases [27,28]. Consequently, AFB1 presents a significant economic challenge for the dairy cattle industry. As a matter of fact, in Europe, AFB1 contamination in animal feed poses a notable risk, with some samples showing a 27% positivity rate, and 18% of samples exceeding the EU’s 5 µg/kg limit allowed in dairy cow feed [29]. Globally, about 25% of food and feed products are contaminated with mycotoxins, with annual U.S. economic losses due to aflatoxin contamination estimated between $20 million and $1.68 billion USD. Regulatory bodies, including the Food and Agriculture Organization (FAO), the World Health Organization (WHO), and the European Union (EU), have implemented stringent AFB1 limits in food and feed to safeguard public health; specifically, the limits for food products range from 0.10 to 12 µg/kg [30,31,32,33]. Notably, when food exceeds regulatory safety thresholds for human consumption, it is often repurposed as animal feed; however, if contamination levels are excessively high, it must be discarded entirely, leading to considerable annual losses in the agricultural industry.

Hence, understanding the genetic variability that influences bovine CYP3A-mediated AFB1 metabolism could provide insights into strategies for mitigating the impact of AFB1 in livestock farming.

In our previous research, we sequenced the *CYP3A* gene cluster in 300 Piedmontese beef cattle samples through deep sequencing and we identified 13 missense single nucleotide variants (SNVs). We then evaluated their functional impact by heterologous expression and subsequent measurement of CYP3A-dependent catalytic activity, using testosterone (TST) and nifedipine (NIF) as CYP3A probe substrates [16].

The present study aims to further investigate how genetic polymorphisms may affect bovine CYP3A protein stability and xenobiotic metabolism. First, we updated the calling and annotation of the genetic variants within the bovine *CYP3A* cluster by analyzing them using a more recent assembly of the bovine genome (i.e., Bos Taurus assembly 9; bosTau9). The updated reference genome (bosTau9) now includes a more complete sequence of the *CYP3A28* gene, specifically capturing the first four exons, which encode the initial (N-terminal) 105 residues of the protein. Previously, the bosTau6 assembly lacked this section, meaning that variants within this protein region could not be detected during variant calling against that version. With the bosTau9 assembly, these variants can now be identified, allowing for a more comprehensive analysis of *CYP3A28* genetic diversity in cattle. Thanks to this approach, we discovered a new missense CYP3A28 variant that was preliminarily characterized in silico using NIF, a channel blocker, well-known as human CYP3A4 probe substrate [16,34]. Then, computational mutagenesis and molecular docking simulations were applied to predict how all these mutations might influence protein stability, as well as the binding and consequently the metabolism of AFB1.

Overall, by identifying mutations that alter CYP3A activity, this research may serve to address public health concerns related to xenobiotic residues in food. Indeed, breeding cattle with favorable genetic traits may improve drug metabolism and reduce toxin bioactivation, thereby improving animal welfare and consumer food safety.

## 2. Results

### 2.1. Missense Variants Identification

In our previous work [16], the variant calling of the raw *CYP3A* sequencing reads from 300 Piedmontese bulls was conducted against the bosTau6 reference genome. Here, we have repeated the variant calling against the more recent assembly of the domestic cattle reference genome, bosTau9. The results of the two variant calling procedures are summarized in Table 1. In the first three columns of Table 1, a summary of the variants identified in [16] on bosTau6 is reported; in the last three columns of Table 1, the variants identified in this study on bosTau9 are presented. For the transcript *CYP3A28*, one variant, c.G931A (p.E311K), which had been identified in [16] as low quality (Quality 39.98), has not been confirmed on bosTau9. In the previous work, besides not having passed the quality filter, this variant was found as heterozygous in only 7 individuals out of 300 by melting curve genotyping. Based on the previous and present results, this variant was therefore considered not present in the studied cohort of Piedmontese bulls.

Conversely, in the updated bosTau9 assembly, we identified the variant c.C313T (p.R105W), which was not detected in our previous work against bosTau6 assembly due to the absence of the exons encoding the first 105 residues. These initial residues span the N-terminal transmembrane domain, essential for anchoring the enzyme to the endoplasmic reticulum. In the protein’s 3D structure, residue ARG105 is located in proximity to the binding pocket (13 Å from the heme iron), suggesting that variants at this site could affect ligand binding and potentially alter enzymatic activity. The newly identified R105W variant is located at position 105 of the protein sequence of transcript ENSBTAT00000016177 on bosTau9. Interestingly, the ENSBTAT00000016177 transcript in bosTau6 lacks the first 105 codons (which are now present on the bosTau9 transcript); therefore, this variant could not be identified before. The remaining six variants previously identified in CYP3A28 [16] have been confirmed here, and are, as expected, shifted by exactly 105 positions along the protein sequence. In summary, a total of seven high-confidence variants have been identified for CYP3A28.

For the other two CYP3A isoforms, CYP3A74 and CYP3A76, all the variants called in [16] are confirmed except for the A289V, which was flagged as a low-quality SNV after filtering in the previous work, and for which the genotyping assay was not successful in detecting the mutation [16].

Finally, we run a computational prediction of variant deleteriousness included in the Ensembl VEP software v.112.0. Of all the SNVs identified in three isoforms, the only variant predicted to have a deleterious effect is R105W in CYP3A28.

### 2.2. Homology Modeling

The homology models for bovine CYP3A28, CYP3A74, and CYP3A76 were generated using the human CYP3A4 structure (PDB: 5TE8) as a template. The sequence identities between the bovine CYP3As and the human template are 71%, 77%, and 77%, respectively. SCRs were identified across all models, and Ramachandran plot analysis confirmed that most of the dihedral angles (φ and ψ) were located in the allowed regions, indicating the structural integrity of the models.

### 2.3. Computational Mutagenesis and Stability Analysis

The mutations were introduced into the wild-type (WT) CYP3A models using the Residue Scanning function of the BioLuminate module (Schrödinger Maestro version 14.1).

The majority of CYP3A28, CYP3A74, and CYP3A76 SNVs identified here are located on the surface of the respective proteins, thus they are far from the binding pocket of the enzyme, as previously described [16]. Only R105W and E374D variants in CYP3A28 and CYP3A76, respectively, are located ~13 Å from the heme.

The stability of the mutated proteins was assessed using the Prime MM-GBSA method, which provided ΔΔ Prime Energy values in kJ/mol. The results are summarized in Table 2.

CYP3A28 isoform displayed a broad range of effects due to SNVs. R105W and N286T caused the most significant destabilization, with ΔΔPrime energy values of 37.32 kJ/mol and 37.84 kJ/mol, respectively. Also, the N422K variant showed a notable destabilizing impact, even if lower than the abovementioned ones. Conversely, H326D, V331L, and V457I variants showed negative ΔΔPrime energy values, indicative of stabilizing effects.

For the CYP3A74 isoform, the G197S and I388V mutations exhibited minimal effects on the stability, with ΔΔPrime energy values of 0.65 kJ/mol and 0.88 kJ/mol, respectively.

In the case of the CYP3A76 isoform, the F376L mutation showed a moderate destabilizing effect (ΔΔPrime energy of 2.54 kJ/mol), while E374D and V253I mutations showed stabilizing effects, reflected by negative ΔΔPrime energy values.

### 2.4. Molecular Docking of NIF into CYP3A28

**Figure 1:**
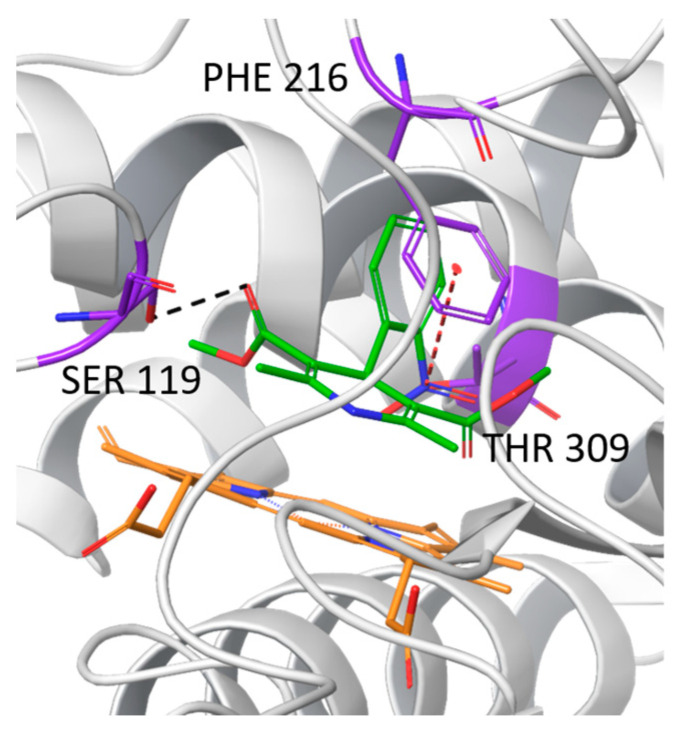
NIF accommodation in the CYP3A28 substrate pocket. Black dashes indicated hydrogen bonds, while red dashes indicated π–cation interaction. The CYP backbone is shown in grey ribbon, the heme group in orange, the residues in violet and NIF in green.

To preliminarily characterize the newly identified R105W mutation, a docking study was performed using NIF as a model CYP3A substrate, as was done in our previous study [16]. Results showed that NIF could be docked into both the WT and mutant CYP3A28 substrate binding pockets, consistent with previous findings [16]. However, the R105W variant, which has never been characterized before, could not accommodate NIF in the substrate pocket. Overall, the docking analysis revealed a hydrogen bond with serine (SER) 119 and a π-cation interaction with phenylalanine (PHE) 216 (Figure 1).

### 2.5. Molecular Docking of AFB1 into CYP3A Isoforms

AFB1 is known to be bioactivated into harmful derivatives (such as the *exo*-epoxide) by CYP3A in many species, including cattle. Therefore, it is of interest to understand whether SNVs in CYP3A could influence the toxicokinetic fate of this mycotoxin.

**Figure 2:**
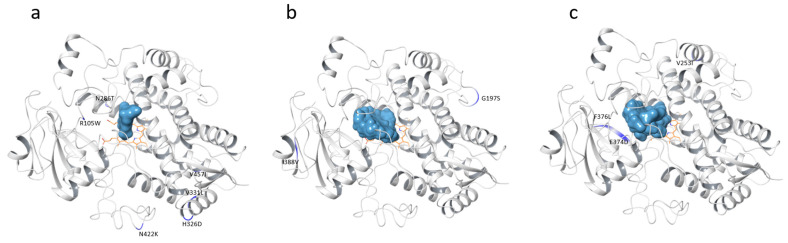
AFB1 (light blue) and variants (blue) location in the CYP3A28 (**a**), CYP3A74 (**b**), and CYP3A76 (**c**) isoforms.

Overall, the docking poses of CYP3A models revealed that AFB1 occupied a large substrate pocket close to the heme iron catalytic center (Figure 2).

**Figure 3:**
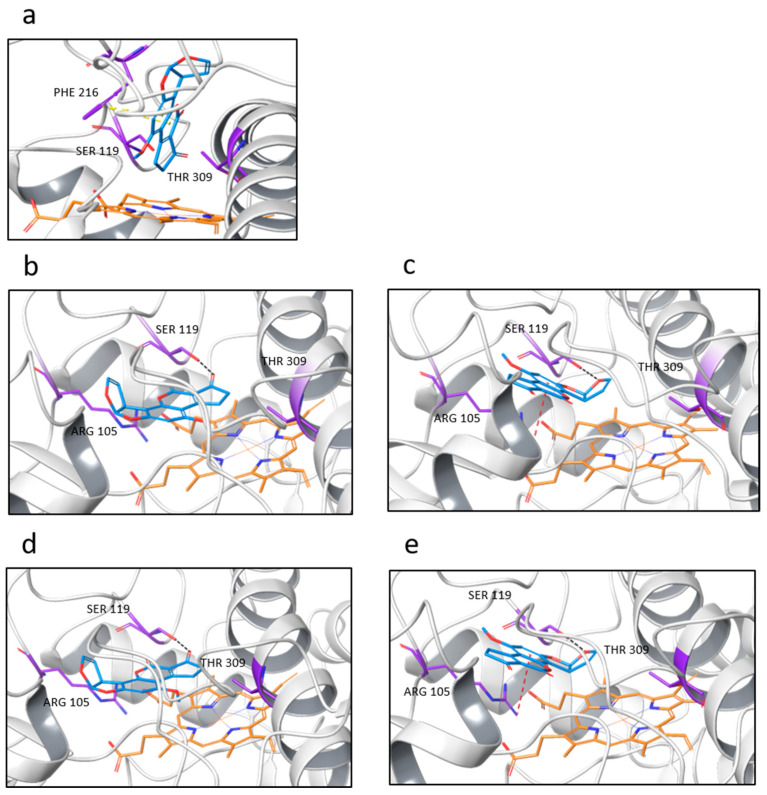
Docking poses of AFB1 against CYP3A28 (**a**), CYP3A74 (**b**,**c**), and CYP3A76 (**d**,**e**) models. CYP backbone is shown in grey ribbon, the heme group in orange, the residues in violet, and AFB1 in blue. Black dashes indicated hydrogen bonds, while yellow and red dashes indicated π–π stacking and π–cation interactions, respectively.

Regarding the CYP3A28 isoform, both WT and mutants may accommodate AFB1 in their binding pocket, with the carbon at position 3 oriented perpendicularly to the heme group (Figure 3a). A closer investigation showed π–π stacking interactions between PHE 216 and rings B and C, while residues such as SER 119 and threonine (THR) 309, which are capable of forming hydrogen bonding interactions, surrounded the binding pocket. Notably, in the case of the R105W variant, no accommodation of the mycotoxin in the binding pocket was observed, suggesting the impact of this genetic modification on AFB1 metabolism, and specifically on AFQ1 production.

Regarding CYP3A74 and CYP3A76, the orientations of the docking poses were quite similar for both WT and mutant models. Specifically, two common types of poses were identified through the in-silico docking analysis (Figure 3b–d). In the first one (Figure 3b,d), the carbon-3 was co-planar with the heme iron catalytic center, with a hydrogen bond between SER 119 and the carbonyl oxygen on the ring A, supporting the formation of AFB1 3α-hydroxy metabolite.

In the second pose (Figure 3c,e), the *exo* side of the double bond between the carbon-8 and carbon-9 faced the heme group, thereby assuming an orientation prone to the formation of the AFB1 8,9-*exo*-epoxide. Specifically, in the second pose, only the WT models and the CYP3A76 mutants exhibited a π–cation interaction between ARG 105 and the ring C, along with a hydrogen bond between SER 119 and the carbonyl oxygen on ring E.

## 3. Discussion

In our previous work [16], we identified thirteen missense SNVs in the CYP3A subfamily of Piedmontese beef cattle through deep sequencing of the *CYP3A* cluster. We then evaluated their functional effects by heterologous expression and measurement of CYP3A-dependent catalytic activity, revealing differences in the metabolism of probe substrates TST and NIF [16].

In the present study, we updated the catalog of genetic mutations within the bovine *CYP3A* gene cluster using the more recent bovine genome assembly bosTau9. Overall, all previously identified variants were confirmed, except for the c.G931A:p.E311K variant, which in the previous variant calling was reported as low quality. Notably, a novel variant, c.C313T:p.R105W, was identified in the CYP3A28 isoform. This discrepancy arises because the ENSBTAT00000016177 transcript in bosTau6 lacks the first 105 codons, which are now included in the corresponding bosTau9 transcript.

This study investigated the structural and functional implications of missense variants in the CYP3A subfamily, with a specific focus on the newly discovered R105W mutation in CYP3A28, and their potential effects on xenobiotic metabolism. Specifically, we focused on AFB1, a mycotoxin of human and animal concern, which is known to be metabolized by CYP3A isoforms [22,23].

Among the analyzed missense variants, the R105W mutation in CYP3A28 stands out for its destabilizing effect, likely due to its location near the enzyme’s substrate entry channel near the binding pocket, as determined through homology modeling based on the structure of human CYP3A4. This mutation replaces a positively charged arginine with a bulky, hydrophobic tryptophan, potentially disrupting local interactions and folding within the binding pocket and impairing the enzyme’s ability to effectively accommodate substrates. Docking studies confirmed this, showing that the R105W mutant could not accommodate NIF, indicating a compromised substrate binding capacity.

The critical positioning of R105 (located 13 Å from the heme iron within the substrate entry channel) underscores its role in orientating and stabilizing substrates as they approach the active site. Indeed, residues in this region are known to play key roles in regulating substrate access and maintaining conformational stability near the binding pocket [35]. Specifically, residues within the entry channel, including position 105, are shown to establish long-range interactions with the heme, guiding substrate orientation upon entry and stabilizing intermediate conformations during the binding process [36]. This role of channel-lining residues is well documented across various CYP450 isoforms, where single-point mutations can markedly impact substrate positioning and binding efficiency. In CYP2C9, the L90P mutation alters the shape of the binding pocket and reduces affinity for hydrophobic substrates, while in CYP2D6, the F120A mutation impacts regioselectivity during substrate oxidation [37,38]. Collectively, these findings underscore how even single-residue mutations in channel-lining regions can drastically modify enzyme specificity and catalytic efficiency.

In addition to R105W, other mutations in CYP3A28, such as N286T and N422K, also destabilize the enzyme’s structure. Although these mutations are distant from the catalytic site and may not directly impact substrate binding, they can still indirectly influence enzyme function. By compromising the structural integrity of the protein, they can induce conformational changes that disrupt the proper orientation and flexibility of the active site, ultimately impairing the enzyme’s ability to function effectively.

Regarding the CYP3A74 and CYP3A76 isoforms, our findings indicated that variants G197S and I388V in CYP3A74, and F376L in CYP3A76 exhibited minimal effects on protein stability, with variant F376L in CYP3A76 resulting in a moderate destabilizing effect. Conversely, variants E374D and V253I in CYP3A76 were associated with stabilizing effects, suggesting that these variants may enhance the enzyme’s functional capacity. In fact, in our previous study [16], heterologous expression of the E374D variant led to a three-fold increase in the production of 16β-OH TST compared to the WT. Worth mentioning, E374D SNV was one of the few variants identified here that was located near the catalytic domain of the enzyme.

The docking analysis of CYP3A74 and CYP3A76 revealed two distinct orientations of AFB1 within the substrate-binding pocket, both showing a hydrogen bond between SER 119 and the mycotoxin. Similarly, a previous study highlighted the crucial role of SER 119 in porcine CYP3A46 in stabilizing AFB1 near the heme catalytic center [39]. SER 119 has a well-established role in substrate binding and orientation within P450 enzymes. Liu and collaborators [36] demonstrated that SER 119 in human CYP3A4 and CYP3A5 enzymes forms a stable, high-occupancy hydrogen bond with substrates such as midazolam, which aids in precise substrate positioning within the active site, enhancing binding affinity. Sevrioukova [40] further illustrated that SER 119 stabilizes interactions with planar polyaromatic molecules, particularly through hydrogen bond formation within the enzyme’s substrate channel. Lewis and colleagues [41] demonstrated via molecular modeling in human CYP3A4 that SER 119 helps in orientating substrates like coumarin within the enzyme’s active site, facilitating 3-hydroxylation. Finally, Xu et al. [42] found that SER 119 forms a polar interaction with citrinin, assisting substrate positioning in the catalytic pocket of human CYP3A4.

This emphasizes the importance of specific amino acids in substrate interaction and supports the hypothesis that the R105W mutation, which replaces a positively charged arginine with a bulky, hydrophobic tryptophan near the substrate-binding pocket, may disrupt similar binding interactions in bovine CYP3A28, potentially impairing AFB1 metabolism.

However, it is important to consider the limitations of this study. The computational approach provides valuable insights into the functional impact of CYP3A mutations, but experimental validation, such as enzyme kinetic assays and in vitro metabolism studies, is necessary to confirm the predicted effects on protein stability and metabolic activity. Our work provides a computational foundation for further experimental validation and contributes to a prioritized ranking of CYP3A variants, guiding future in vitro studies on their functional impacts.

## 4. Materials and Methods

### 4.1. Variant Calling and Annotation

The sequencing of the *CYP3A* cluster in a cohort comprising 300 Piedmontese bulls was previously detailed in [16]. However, while the previous study [16] aligned reads against the bosTau6 reference genome, this study utilized the bosTau9 (ARS-UCD1.2) reference genome, downloaded from the UCSC Golden Path database (https://hgdownload.soe.ucsc.edu/goldenPath/bosTau9/bigZips/bosTau9.fa.gz (accessed on 3 February 2024)). FASTQ reads were (i) filtered for adapter contaminations and low-quality reads with Trimmomatic v.0.39, (ii) aligned to the bosTau9 reference using BWA-MEM with default parameters, and (iii) flagged duplicate reads in each BAM file using the Picard (v.2.27.5) MarkDuplicates tool embedded in the genome analysis tool kit (GATK v.4.3.0.0), (iv) modified read groups using Picard (v.2.27.5) AddOrReplaceReadGroups, (v) applied the BaseRecalibrator and ApplyBQSR functions of GATK v.4.3.0.0 for base quality score recalibration of each BAM file. Variant calling was performed with the GATK v.4.3.0.0 HaplotypeCaller [43] and the variants were annotated using Ensembl VEP v.112.0 [44].

### 4.2. Homology Modeling and Computational Mutagenesis of CYP3As

Homology modeling and computational residue mutations were performed using Schrödinger Maestro version 14.1 (Small-Molecule Drug Discovery Suite 2024-3, Schrödinger, LLC, New York, NY, USA).

To build the bovine WT CYP3A models (i.e., CYP3A74, CYP3A76 and CYP3A28), the crystallized human CYP3A4 bound to midazolam (PDB: 5TE8, R = 2.55 Å) [45] was used as the template. The template was first prepared using the Protein Preparation Wizard tool. Specifically, bond orders and hydrogens were added, and missing chains and loops were filled using the Prime module. Chain A was used in the study, and heteroatom states were generated using Epik (pH 7 ± 2). The heme iron was set as Fe3+ and connected via zero-order bonds to the conserved cysteine sulfur and the four heme nitrogens. Hydrogen bonds were assigned using PROPKA (pH 7.0), and water molecules with fewer than three hydrogen bonds to non-water molecules were removed using default parameters. Protein minimization was conducted using the OPLS2005 force field until the heavy-atom displacement converged to a root mean square deviation (RMSD) of 0.30 Å.

For homology modeling, sequence alignment was performed using the Smith–Waterman algorithm in the Multiple Sequence Viewer/Editor. The Build Homology Model tool was then implemented to obtain the 3D models. Structurally Conserved Regions (SCRs) were checked, and a Ramachandran plot was constructed to evaluate the quality of the obtained models.

The mutations were introduced into the WT CYP3As using the Residue Scanning module of BioLuminate. To evaluate the stability of the mutated proteins, the Prime MM-GBSA (Molecular Mechanics Generalized Born Surface Area) method was employed [46].

### 4.3. Molecular Docking of NIF and AFB1 into WT and Mutated CYP3A Models

The molecular docking studies of NIF and AFB1 were performed using Schrödinger’s Small-Molecule Drug Discovery Suite to evaluate their interactions with both WT and mutated CYP3A models. NIF and AFB1 (PubChem CID: 4485 and 186907, respectively) were prepared using the LigPrep module. The ligands were ionized at a pH of 7 ± 2, and desalted and tautomeric forms were generated using Epik. The ligand structures were minimized using the OPLS4 force field to ensure accurate geometry and energetics.

Substrates were docked into the CYP models using the Glide in Standard Precision (SP) mode. Specifically, a grid approximately 20 Å in size was centered on the substrate binding pocket and the heme porphyrin ring. Molecular docking was performed with a flexible ligand in search of the optimal conformation in the rigid binding pocket.

## Figures and Tables

**Table 1:** List of missense variants identified in the cattle CYP3A isoforms.

Variants Identified on bosTau6 [16]			Variants Identified on bosTau9		
Position (bp) (bosTau6)	cDNA SNV (bosTau6 Transcript)	NCBI ID	Position (bp) (bosTau9)	cDNA SNV (bosTau9 Transcript)	NCBI ID
*CYP3A28*					
N.A.	N.A.	N.A.	25:36561518	**ENSBTAT00000016177** **c.C313T(p.R105W)**	**rs447850593**
25: 37031758	ENSBTAT00000016177 c.A539C(p.N180T)	rs384714392	25:36543302	ENSBTAT00000016177 c.A**857**C(p.N**286**T)	rs384714392
25: 37037420	ENSBTAT00000016177 c.C658G(p.H220D)	rs381315842	25:36537640	ENSBTAT00000016177 c.C**976**G(p.H**326**D)	rs381315842
25: 37037435	ENSBTAT00000016177 c.G673C(p.V225L)	rs110013281	25:36537625	ENSBTAT00000016177 c.G**991**C(p.V**331**L)	**rs3423551575**
25: 37040096	ENSBTAT00000016177 c.G931A(p.E311K)	N.A.	N.A.	N.A.	N.A.
25: 37042300	ENSBTAT00000016177 c.T948G(p.N316K)	rs384081812	25:36532760	ENSBTAT00000016177 c.T**1266**G(p.N**422**K)	rs384081812
25: 37042403	ENSBTAT00000016177 c.G1051A(p.V351I)	rs137124349	25:36532657	ENSBTAT00000016177 c.G**1369**A(p.V**457**I)	**rs3423517093**
25: 37044274	ENSBTAT00000016177 c.G1172A(p.G391D)	rs384367918	25:36530786	ENSBTAT00000016177 c.G**1490**A(p.G**497**D)	rs384367918
*CYP3A74*					
25: 37150701	ENSBTAT00000063483 c.G589A(p.G197S)	rs384467435	25: 36609181	ENSBTAT00000007170 c.G589A(p.G197S)	rs384467435
25: 37135166	ENSBTAT00000063483 c.C866T(p.A289V)	rs433125080	N.A.	N.A.	N.A.
25: 37131297	ENSBTAT00000063483 c.A1162G(p.I388V)	rs454167819	25: 36589780	ENSBTAT00000007170 c.A1162G(p.I388V)	rs454167819
*CYP3A76*					
25: 37202239	ENSBTAT00000007170 c.G757A(p.V253I)	rs440751676	25: 36660502	ENSBTAT00000063483 c.G757A(p.V253I)	rs440751676
25: 37194823	ENSBTAT00000007170 c.G1122T(p.E374D)	N.A.	25: 36653085	ENSBTAT00000063483 c.G1122T(p.E374D)	N.A.
25: 37194819	ENSBTAT00000007170 c.T1126C(p.F376L)	N.A.	25:36653081	ENSBTAT00000063483 c.T1126C(p.F376L)	N.A.

In columns 1, 2, and 3, the variants identified on the bosTau6 reference genome as published in [16] are shown. In columns 4, 5, and 6, the variants identified in this study on the bosTau9 reference genome are shown. The grey background and bold font highlight differences between the results of the variant calling on the bosTau6 in comparison to the bosTau9 reference genome.

**Table 2:** ΔΔPrime Energy values for each single nucleotide variant (SNV) of CYP3A isoforms.

CYP3A Isoform	SNV	Prime Energy (kJ/mol)
CYP3A28	R105W	37.32
	N286T	37.84
	H326D	−28.4
	V331L	−17.85
	N422K	28.58
	V457I	−4.23
CYP3A74	G197S	0.65
	I388V	0.88
CYP3A76	V253I	−0.38
	E374D	−1.44
	F376L	2.54

## Data Availability

Data is contained within the article.
